# HA2-FimA DNA Vaccine Treats Experimental Periodontitis

**DOI:** 10.3290/j.ohpd.b5281939

**Published:** 2024-04-30

**Authors:** Huijie Zhang, Yueyue Wang, Zhu Wang, Nanqing Fu, Xinrui Wang, Guohui Bai

**Affiliations:** a Postgraduate Student, School of Stomatology, Zunyi Medical University, Key Laboratory of Oral Disease Research, School of Stomatology, Zunyi Medical University, China. Study design, data analysis and interpretation, drafted and critically revised the manuscript.; b Dentist, Hospital/School of Stomatology, Zunyi Medical University, Key Laboratory of Oral Disease Research, School of Stomatology, Zunyi Medical University, China. Study design, data analysis and interpretation.; c Postgraduate Student, School of Stomatology, Zunyi Medical University, Key Laboratory of Oral Disease Research, School of Stomatology, Medical University, China. Studied animal transcriptomic sequencing, analysed and processed the sequencing results.; d Postgraduate Student, School of Stomatology, Zunyi Medical University, China. Studied animal transcriptomic sequencing, analysed and processed the sequencing results.; e Postgraduate Student, School of Stomatology, Zunyi Medical University, Key Laboratory of Oral Disease Research, School of Stomatology, Zunyi Medical University, China. Analysed and visualised data of alveolar bone resorption.; f Professor, School of Stomatology, Zunyi Medical University, Key Laboratory of Oral Disease Research, School of Stomatology, Zunyi Medical University, China. Research design, data interpretation, critical revision of manuscript.

**Keywords:** CAMP, DNA vaccine, SIgA

## Abstract

**Purpose:**

To study the therapeutic effect of hemagglutinin-2 and fimbrial (HA2-FimA) vaccine on experimental periodontitis in rats.

**Materials and Methods:**

The first batch of rats was divided into two groups and immunised with pure water or pVAX1-HA2-FimA at the age of 6, 7, and 9 weeks. After sacrificing the animals, total RNA was extracted from the spleens for RNA high-throughput sequencing (RNA-Seq) analysis. The second batch of rats was divided into four groups (A, B, C, D), and an experimental periodontitis rat model was established by suturing silk thread around the maxillary second molars of rats in groups B, C, and D for 4 weeks. The rats were immunised with pure water, pVAX1-HA2-FimA vaccine, empty pVAX1 vector, and pure water at 10, 11, and 13 weeks of age, respectively. Secretory immunoglobulin A (SIgA) antibodies and cathelicidin antimicrobial peptide (CAMP) levels in saliva were measured by enzyme-linked immunosorbent assay (ELISA). All rats were euthanised at 17 weeks of age, and alveolar bone loss was examined using micro-computed tomography (Micro-CT).

**Results:**

Through sequencing analysis, six key genes, including Camp, were identified. Compared with the other three groups, the rats in the periodontitis+pVAX1-HA2-FimA vaccine group showed higher levels of SIgA and CAMP (p < 0.05). Micro-CT results showed significantly less alveolar bone loss in the periodontitis+pVAX1-HA2-FimA vaccine group compared to the periodontitis+pVAX1 group and periodontitis+pure water group (p < 0.05).

**Conclusion:**

HA2-FimA DNA vaccine can increase the levels of SIgA and CAMP in the saliva of experimental periodontitis model rats and reduce alveolar bone loss.

Periodontitis is a common chronic inflammatory disease characterised by gum inflammation, attachment loss, periodontal pocket formation, and alveolar bone resorption. Prolonged inflammation can result in tooth mobility and loss.^9^ Numerous risk factors are associated with periodontitis. Current research suggests that periodontitis is primarily caused by dysbiosis of subgingival microbial communities, which have detrimental effects on the host immune system, leading to the establishment and maintenance of unmitigated inflammation in gingival and periodontal tissues, thereby preventing immune subversion and tissue recovery.^2,6^ Key species in oral biofilm include *Porphyromonas gingivalis* (*P. gingivalis*), *Tannerella forsythia* (*T. forsythia*) and *Treponema denticola* (*T. denticola*); these are recognised as aetiological agents in the development of periodontal disease.^15^ Due to the ability of *P. gingivalis* to interact with the host’s innate responses and persist in the periodontal pocket, it is considered a “keystone” pathogen in the dysbiosis of subgingival biofilms.^16^

*P. gingivalis* possesses various potential virulence factors, such as hemagglutinins, gingipains, fimbriae, encapsulation, and lipopolysaccharides, which contribute to its evasion, destruction of host immunity, and invasion of host periodontal tissues.^5^ Due to the lack of enzymes required for heme synthesis, *P. gingivalis* degrades host hemoglobin through hemagglutinin-2 (HA2) to obtain the necessary porphyrins and trivalent iron for its growth and virulence. Therefore, inhibiting the binding activity between *P. gingivalis* and hemoglobin is advantageous in reducing its virulence.^1^ Fimbriae play a crucial role in the initial attachment and colonisation process of *P. gingivalis*. Research has shown that *P. gingivalis* expresses two different types of fimbriae in its extracellular membrane: long fimbriae encoded by the fimA gene and short fimbriae encoded by the mfa1 gene.^8^ Besides aiding in host adhesion, long fimbriae also mediate co-aggregation with other oral pathogens such as *T. denticola*, *Streptococcus oralis* (*S. oralis*), and *Streptococcus gordonii* (*S. gordonii*).^15^ Therefore, these two virulence factors mentioned above have the potential to serve as candidate vaccine antigens.

In this study, by obtaining the virulence factors HA2 and FimA of *P. gingivalis*, we constructed a periodontitis gene vaccine and immunised SD rats with mucosal vaccine. In order to investigate the mechanisms of periodontitis gene vaccines at the molecular level, this study utilised RNA-seq technology to collect gene expression data between different groups. RNA sequencing (RNA-seq) is a technique used to quantify gene expression levels and gene expression patterns. It is widely used to uncover genomic changes between transcriptome samples.^19^ Gene Ontology, Kyoto Encyclopedia of Genes and Genomes and protein-protein interaction were used to analyse the data, and key genes were selected for further research. This study aimed to explore the mechanism of periodontitis gene vaccines and provide a theoretical basis for optimising future vaccines.

## Materials and Methods

### Laboratory Animals

This experiment used two batches of male Sprague Dawley rats (SD rats) at four weeks of age (100 ± 20 g). All rats were fed a standard diet and kept in a room with a temperature of 24 ± 2°C, relative humidity of 40%~70%, and a 12-h light-dark cycle. The experiment was conducted in a pathogen-free environment under clean and ventilated conditions following one week of acclimation. The first batch of rats was randomly divided into group A (control group) and group B (pVAX1-HA2-FimA vaccine). The second batch of rats was randomly divided into four groups: group A (blank), group B (periodontitis + pVAX1-HA2-FimA vaccine), group C (periodontitis + pVAX1 empty vector), and group D (periodontitis + pure water). The animal experiment was conducted with the approval of the Animal Ethics Committee of Zunyi Medical University and complied with all relevant institutional and governmental regulations regarding the ethical use of animals (Animal Ethics Review Number: [2021] 2-009).

### Building the Periodontitis Vaccine

The total DNA of *P. gingivalis* W83 was extracted by our research group. The virulence factors HA2 and FimA of *P. gingivalis* were obtained, and the HA2, FimA, and IRES sequence genes were spliced to obtain the HA2-FimA fragment. A periodontitis gene vaccine was constructed using pIRES2-EGFP as a template and pVAX1 plasmid as a vector. The recombinant plasmid was then transferred into DH5α-competent cells, and positive recombinants were selected for sequencing, amplification, and preservation. The preserved strains were stored in glycerol in a freezer at -80°C and maintained at the Key Laboratory of Oral Diseases Research of Ordinary Institutions of Higher Learning in Guizhou Province, China.

### Strain Recovery and Culture

The glycerol stock containing pVAX1-HA2-FimA was thawed, and a sterile inoculation loop was used to streak the bacteria onto LB-kana solid medium. The plate was then incubated at 37°C for 16 h. A single colony from the LB-kana solid medium was picked out using an inoculation loop and transferred to 100 ml of LB-kana liquid medium. The culture was incubated at 37°C in a shaking incubator at 180 rpm for 16 h. The bacterial culture was subjected to PCR and double enzyme digestion for verification. Positive clones of pVAX1-HA2-FimA were selected and sent for sequencing at Shenggong Biotechnology (Shanghai, China). The pVAX1-HA2-FimA plasmid was extracted using the Endotoxin-free Plasmid Extraction Kit (Type DP11, TianGen Biotech; Beijing, China), followed by lyophilisation using a freeze-dryer (Telstar; Barcelona, Spain). The concentration was adjusted to 1000 μg/ml with pure water using a microvolume spectrophotometer (Allsheng, China), and the plasmid was aliquoted and stored at -80°C for future use.

### Immunisation of the First Batch of Rats

The first batch of rats (N = 6) was randomly divided into two groups: group A (control) and group B (pVAX1-HA2-FimA vaccine). The rats in the first batch were labeled A1, A2, A3, B1, B2, and B3 according to their respective groups. At 6, 7, and 9 weeks of age, the rats were anesthetised using isoflurane (R510-22-10, RWD Life Science; Shenzhen, China) and immunised through intranasal instillation. Immunisation was performed by alternately instilling 100 µl of the vaccine dose into the bilateral nasal cavities using a micro-pipette. The plasmid concentration used for immunisation was 1000 μg/ml.

### Transcriptomic Sequencing

At the 12th week, all rats were euthanised via cervical dislocation under anesthesia with isoflurane. Spleen tissue was collected from all rats. Total RNA from the spleen tissues of the two groups was extracted using the RNA Extraction Kit (Easy Fast Animal Tissue/Cell Total RNA Extraction Kit DP451, TIANGEN; Beijing, China). Preliminary detection was performed using agarose gel electrophoresis, and the concentration and integrity of the extracted RNA were measured using a microvolume spectrophotometer and an Agilent 2100 Bioanalyser (Agilent Technologies; Santa Clara, CA, USA).

Equal amounts of RNA from both groups of rats were used to construct transcriptome libraries using the NEBNext Ultra RNA Library Prep Kit (Novogene; Beijing, China). mRNA was enriched from total RNA using magnetic beads, and fragmented mRNA was used as a template to synthesise complementary DNA (cDNA) followed by PCR amplification.^24^ Qualified samples were subjected to sequencing using the IlluminaNovaseq 6000 platform, with paired-end reads and a sequencing strategy of PE150. After sequencing, the data were quality-controlled using Fastp version 0.19.7 (OpenGene/GitHub.com).

The raw data were filtered to remove reads with adapter contamination, reads with undetermined bases, and low-quality reads (sequencing base quality ≤20).^21^ The filtered data were normalised using DESeq2 v.1.16.1 for differential expression analysis. This software uses a negative binomial distribution model to detect differentially expressed genes, with the criteria of a Benjamini & Hochberg adjusted p-value less than 0.05 and |log2FC| greater than 0. GO and KEGG analyses were performed using ClusterProfiler, and the resulting p-values were adjusted using the Benjamini & Hochberg method. The differentially expressed genes were uploaded to the Search Tool for the Retrieval of Interacting Genes (STRING, https://string-db.org/) to obtain the interaction network with a minimum interaction score greater than 0.4. The network was visualised and analysed using Cytoscape v.3.9.0 and the CytoHubba plugin. Integrative analysis of GO, KEGG, and the PPI network was performed to identify potential candidate genes and signaling pathways related to the mechanism of the periodontitis gene vaccine.

The mRNA expression of potential candidate genes was validated by qRT-PCR using the PerfectStart Green qPCR SuperMix (TransGen Biotech). The detailed primer designs are listed in Table 1. The reaction program consisted of step 1: 94°C for 30 s; step 2: 94°C for 5 s; step 3: 60°C for 15 s; step 4: 72°C for 10 s. Steps 2-4 were repeated for 40 cycles. Each sample had three biological replicates, and the mRNA relative expression levels of the target genes were normalised using the β-Actin reference gene.

**Table 1:** Primer sequence table

Primer name	Forward primer (5 ‘- 3’)	Reverse primer (3 ‘- 5’)
β-Actin	TTTCCAGCCTTCCTTCCT	CAGGTCTTTGCGGATGTC
Cd28	GGAGGCAGGAGGATTACCAGGAG	GGAGTTGAGGCAAGGTGTCACAG
Cd3e	ATGACCAACACTGACCAACCAATCC	GGGAGGAGAGGAGGAGGTAGGAG
Elane	GCCTCTGACATTGATGCCTCCAC	AGAATCCAGATCCACAGCCTCCTC
Camp	AGTTGTGATGCGGTGAGTGGAATG	GCTCTCTCTTCTGCTCTGGGTAGG
Defa11	ACTCACTCTGCTCACCACCCTT	ACTCACTCTGCTCACCACCCTT
Mpo	AGGTCAATCGCAGTGGCTTCAAG	CGAGCCTACAGCAACAAGCAGAG

### Establishment and Identification of the Second Batch of Rat Periodontitis Models

The second batch of 52 rats at 4 weeks of age were randomly divided into four groups: A, B, C, and D, with 13 rats in each group. After one week of adaptation feeding, the rats in groups A, B, C, and D were anesthetised in the fifth week by intraperitoneal injection of 2% sodium pentobarbital at a dose of 40 mg/kg. After successful anesthesia, the rats were placed supine, and their limbs and heads fixed. The gingiva and oral mucosa of the rats were observed to confirm oral health. In groups B, C, and D, bilateral maxillary second molars of SD rats were ligated with 4-0 silk sutures. Three loops were made on the buccal side of the rats, and the sutures were gently pressed into the gingival sulcus. The gingival status, tooth mobility, and suture detachment were observed weekly, and if the sutures detached, they were re-ligated. After 4 weeks of suture ligation, one rat was randomly selected from each of the A, B, C, and D groups and euthanised using cervical dislocation under anesthesia with isoflurane. The maxillary bones were dissected.

The bilateral maxillary bones were fixed in 4% tissue cell fixative for 24 h. The maxillary bone samples were scanned using the Hiscan XM Micro CT (Hiscan; Jiangsu, China) with the following scanning conditions: 60 kV, 134 uA, single exposure time of 50 ms, scanning resolution of 50 μm, and scanning angle interval of 0.5 degrees. The scanning data were imported into the Hiscan Analyzer Software to reconstruct a 3D model of the maxillary bone, and measure and analyse the level of alveolar bone resorption in the rat maxilla. After scanning, the maxillary bone samples were soaked in JYBL-II decalcifying solution for 24 h. The periodontal tissue pathology was observed after dehydration, embedding, sectioning, HE staining, and coverslipping.

### Immunisation and Sampling of the Second Batch of Rats after Successful Modeling

After successfully establishing the periodontitis model, the silk threads were removed. Anesthesia was induced in rats aged 10, 11, and 13 weeks using isoflurane, and immunisation was performed through intranasal instillation. The four groups, A, B, C, and D, were respectively administered distilled water, pVAX1-HA2-FimA vaccine, pVAX1 empty vector, and distilled water via intranasal instillation. Each rat received an immunisation dose of 100 µl with a plasmid concentration of 1000 μg/ml.

**Fig 1:**
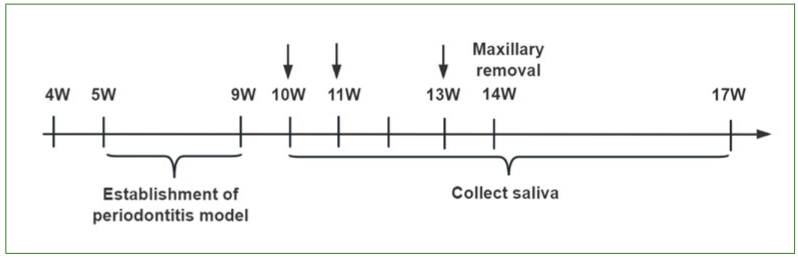
Experimental schedule.

Subcutaneous injection of 0.2% pilocarpine eye drops (0.2 mg/100 g) was performed in the neck region of rats, and approximately 1 minute later, saliva samples of 0.5 ml were collected from the A, B, C, and D groups. The samples were centrifuged at 4°C and high speed (12,000 rpm/10 min) to remove impurities and then stored in new EP tubes at -80°C for future use. Saliva samples were collected from the 9th to the 17th week of rat age, with a total collection period of 9 weeks. The detailed time points of sample collection are shown in Fig 1.

### Enzyme-linked Immunosorbent Assay (ELISA)

The optimal coating concentrations of HA2 and FimA antigens and the optimal dilution concentrations of rat saliva and horseradish peroxidase-conjugated goat anti-rat IgA were determined using a serial dilution method (Table 2).

**Table 2:** Concentration table of salivary cross-dilution method

Antigen name	Optimal dilution of saliva	Optimal coating concentration of antigen	HRP goat anti-rat IgA
HA2 FimA	1:16 1:4	10 µg/ml 1.25 µg/ml	1:1000 1:1000

HRP: horseradish peroxidase conjugated.

The HA2 and FimA antigen concentrations were determined using a BCA assay kit (Beyotime; Shanghai, China). The antigens were diluted in ELISA coating buffer (Solarbio; Beijing, China) with a 10 µg/ml concentration for HA2 and 1.25 µg/ml for FimA. The antigens were added to the ELISA plate, 100 µl per well, and incubated at 4°C for 16 h. To prepare the 1x PBST wash buffer, 10x PBST buffer (Solarbio) was diluted with pure water. 250 µl of 1x PBST was added to each well, let stand for 1 min, after which the plate was washed five times. 5% bovine serum albumin blocking solution (Solarbio) was added to the plate, 200 µl per well, and incubated at 37°C in a humidified chamber for 2 h. The plate was then washed five times. The horseradish peroxidase-conjugated goat anti-rat IgA was diluted in antibody dilution buffer (1:1000), incubated at 37°C in a humidified chamber for 2 h, then the plate was washed five times. Equal volumes of color reagent A and color reagent B from the two-component colorimetric kit (Solarbio) were mixed. Then, 100 µl of the mixed A and B color reagents were added to each well. The plate was wrapped with aluminum foil to protect it from light and incubated at 37°C for 15 min. After colour development, 50 µl of ELISA stop solution was added. The absorbance at 450 nm wavelength was measured within 15 min using a microplate reader.

The CAMP levels in saliva were measured using a Rat Antimicrobial Peptide ELISA kit (Jingmei Biotechnology; Jiangsu, China) following the detailed instructions provided in the kit manual.

### Micro-CT of Rat Alveolar Bone

In the 17th week, bilateral maxillary bone samples were obtained after euthanising four groups of rats using cervical dislocation under isoflurane anesthesia. The samples were fixed in 4% paraformaldehyde for 24 h and then sent to Suzhou Hisfield Information Technology (Suzhou, Jiangsu, China) for scanning using the Hiscan XM Micro CT. 3D reconstruction of the maxillary bone samples was performed, and the level of alveolar bone resorption in the rat was analysed. The maxillary bone samples of the rats euthanised in the 17th week were scanned using Hiscan XM Micro CT. After 3D reconstruction, the distances between the cementoenamel junction and the alveolar crest were measured at six sites (buccal and palatal sides) of the maxillary second molar (mesial, central, and distal). Each site was measured three times, and the mean value was calculated.

### Statistical Analysis

The data were analysed using SPSS 18.0 software (Armonk, NY, USA). For normally distributed data, an independent-samples t-test was used, while for non-normally distributed data, the Wilcoxon rank-sum test was employed. A significance level of α=0.05 was used, and p<0.05 was considered statistically significant. Normally distributed and nearly normally distributed data were presented as mean ± standard deviation, and ANOVA was used for comparing differences among multiple samples. For data that met the assumptions of normality and homogeneity of variances, post-hoc multiple comparisons were conducted using LSD analysis. For data that met the assumption of normality but violated the assumption of homogeneity of variances, Tamhane’s T2 analysis was performed. Kruskal-Wallis analysis was used for non-normally distributed data.

## Results

### Transcriptome Sequencing

Detection was performed using a microplate spectrophotometer and an Agilent 2100 Bioanalyser. The results showed that the total amount of rat samples in groups A and B was > 40 μg, RNA concentration > 400 ng/µl, RIN value ≥ 8.1, and the 28S:18S ratio was within the range of 0.9 to 1.9. The above test results indicate that the extracted total RNA is not statistically significantly degraded and has good integrity and high purity, making it suitable for subsequent transcriptome sequencing.

To investigate the mechanism of the periodontitis gene vaccine pVAX1-HA2-FimA, sequencing was performed on a total of 6 samples (from group-A and -B rats A1, A2, A3, B1, B2, and B3), and transcriptome libraries were constructed. On average, group A and group B generated 49,200 and 46,500 raw reads, respectively. After filtering out low-quality reads and adapter sequences from the raw data, the average number of clean reads were 46,700 and 44,100, respectively. The base alignment rate of the sequencing samples ranged from 94.52% to 95.60%, and the multiple alignment rate ranged from 6.61% to 7.71%. The average ratio of clean reads in groups A and B was 90.24% and 89.36%, respectively. The average Q30% value of clean reads was 94.72% and 94.64%, respectively. The average GC content was 50.45% and 50.06%, respectively.

**Fig 2:**
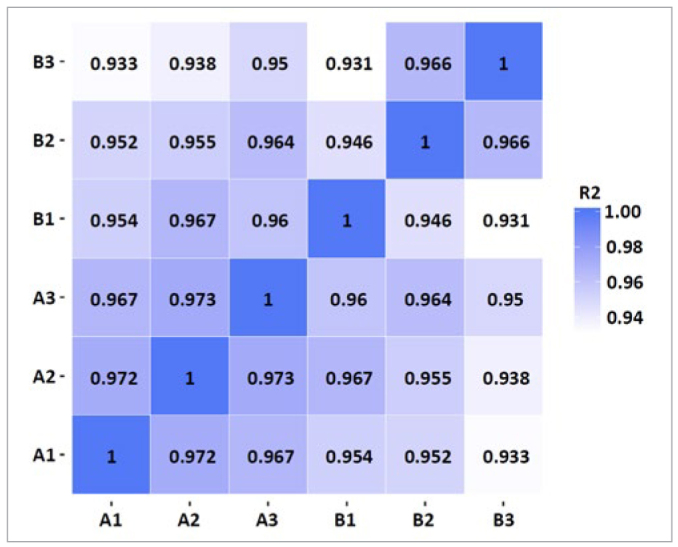
Correlation coefficients of groups A and B. Group A: control group (N = 3, A1, A2, A3); group B: vaccine group (N = 3, B1, B2, B3).

**Fig 3:**
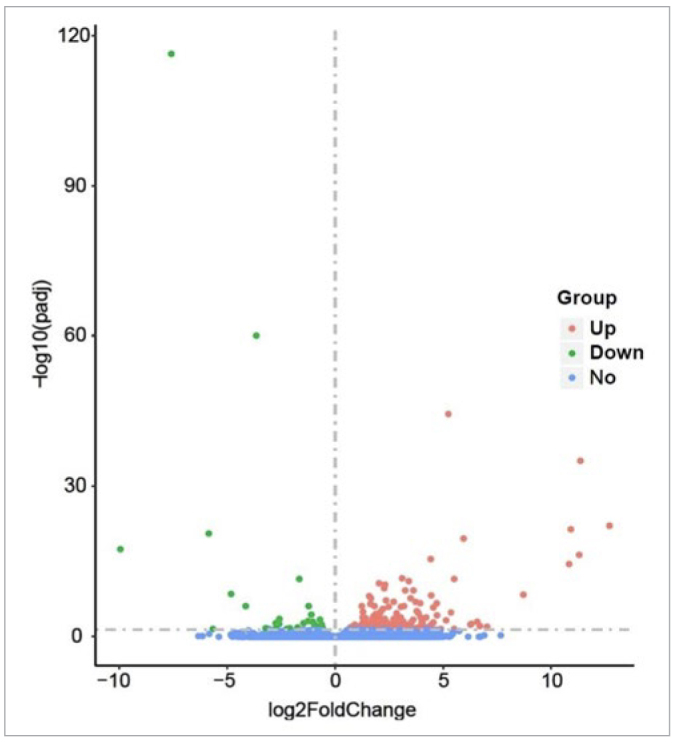
Volcano map of differentially expressed genes of groups A and B. Up: up-regulated gene expression; Down: down-regulated gene expression; No: non-significantly differentially expressed gene.

Before analysing the data, biological replicates of the sequencing samples were examined. The analysis showed that the two sample groups’ correlation coefficients were higher than 0.9 (Fig 2). According to the negative binomial distribution model, the vaccine group had 166 upregulated genes and 52 downregulated genes (Fig 3), and the differences in gene expression between the two groups were statistically significant.

### GO Enrichment Analysis

**Fig 4:**
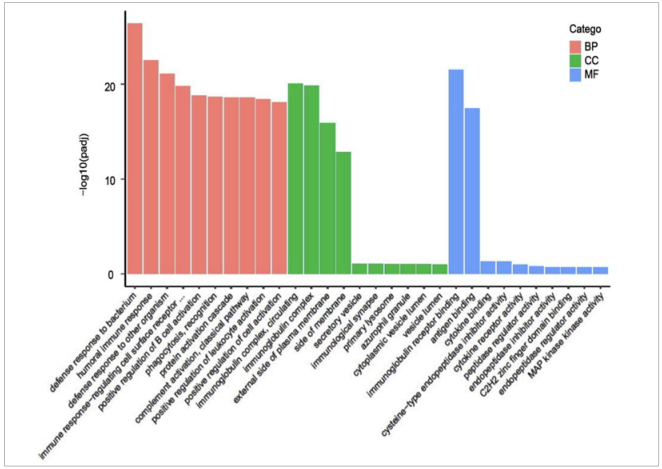
Histogram of GO enrichment analysis of the vaccine and control groups. BP: biological process; CC: cellular component; MF: molecular function.

The differentially expressed genes were subjected to GO functional annotation and enrichment analysis, which included three categories: Biological Process (BP), Cellular Component (CC), and Molecular Function (MF). The top 30 significantly enriched functions were selected and plotted in a bar graph (Fig 4). The results showed that the majority of genes in these three functional categories were involved in immune processes, such as bacterial defense response, humoral immune response, antimicrobial peptide-mediated antimicrobial humoral response, defense response to Gram-negative bacteria, etc. These biological processes may contribute to alleviating the damage to periodontal tissues caused by experimental periodontitis.

### KEGG Enrichment Analysis

**Fig 5:**
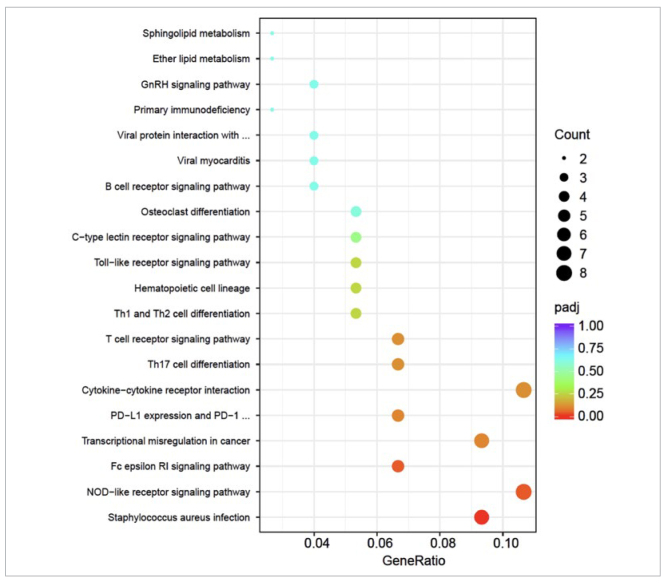
Bubble map of KEGG enrichment analysis in vaccine and control groups.

The top 20 enriched pathways of the differentially expressed genes were analysed using the KEGG pathway enrichment analysis (Fig 5). The results showed statistically significant changes in the NOD-like receptor signaling pathway, *Staphylococcus aureus* infection, and FcεRI signaling pathway. Among them, the NOD-like receptor signaling pathway is closely related to immune processes. In the NOD-like receptor signaling pathway, the gene Mapk11 was statistically significantly downregulated, while the genes Camp, Nlrp12, Defa5, Np4, Defa11, and RatNP-3b were statistically significantly upregulated. One study^10^ has shown that the NOD-like receptor signaling pathway is a key factor in mediating innate immunity. NOD-like receptors can trigger the assembly of inflammasomes and regulate inflammation by affecting the nuclear factor kappa-B (NF-κB) pathway and the mitogen-activated protein kinase (MAPK) pathway.^10^

### Construction and Analysis of the PPI Network Interaction Map

**Fig 6:**
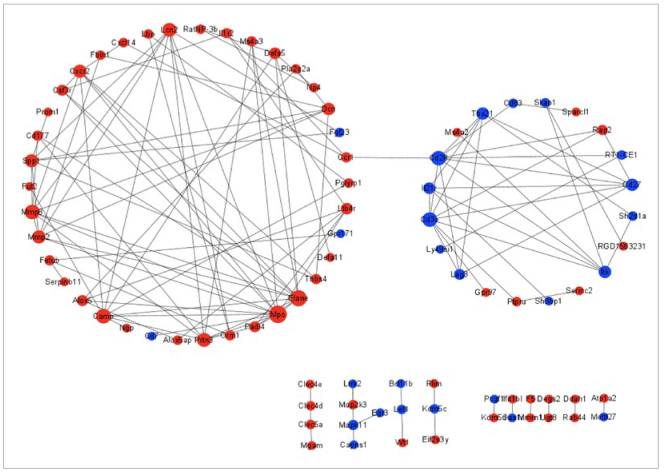
Interaction of statistically significantly different gene networks. The size of the circular node is related to the contribution degree. The larger the circular node is, the higher is the contribution degree. Red nodes indicate up-regulated proteins, and blue nodes indicate down-regulated proteins.

The differentially expressed genes based on the aforementioned filtering criteria were mapped to the STRING database. The exported file was uploaded to Cytoscape software to construct a PPI network graph of these genes. The graph consisted of 85 nodes and 126 edges (Fig 6). The CytoHubba plugin in Cytoscape software was utilised to identify central proteins. The top ten nodes with the highest degree are MPO, ELANE, MMP8, CD3E, CD28, CAMP, PRTN3, LCN2, SPP1, and MMP2. Considering the GO, KEGG, and PPI network analysis results, we selected Cd28, Cd3e, Elane, Camp, Defa11, and Mpo as candidates for statistically significantly different genes.

### Expression of Key Genes after Nasal Drip Immunisation in Rats

**Fig 7:**
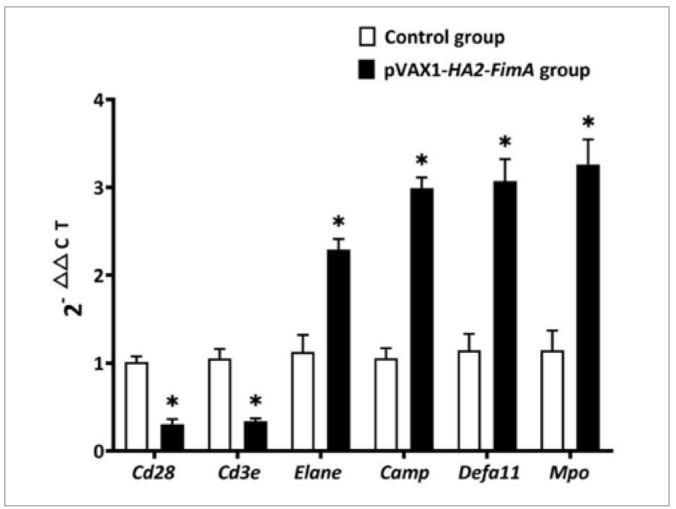
Expression of differentially expressed genes in spleen. *Statistically significant,p < 0.05.

This study used qRT-PCR to validate the selected statistically significantly differently expressed genes and determine the changes in gene expression levels. The qRT-PCR results showed that compared to the control group, the genes Elane, Camp, Defa11, and Mpo were statistically significantly upregulated, while the genes Cd28 and Cd3e were statistically significantly downregulated in the pVAX1-HA2-FimA vaccine group (Fig 7), with p < 0.05. These experimental results are consistent with the transcriptome sequencing results.

### Experimental Periodontitis Model

**Fig 8:**
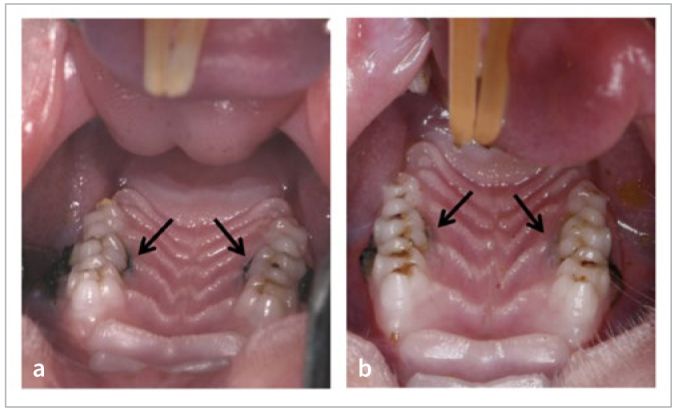
Establishment of experimental periodontitis model in rats. a: The first week of model building; b: Week 4 of model building. Arrow indicates ligature thread.

**Fig 9:**
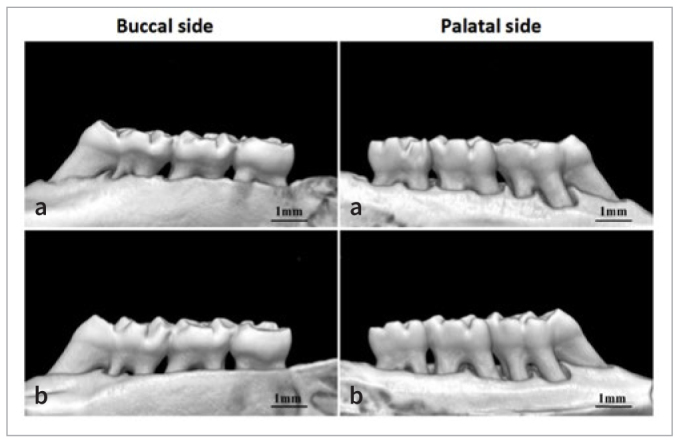
3D reconstruction of alveolar bone of maxillary second molar. a: blank group; b: periodontitis group.

**Fig 10:**
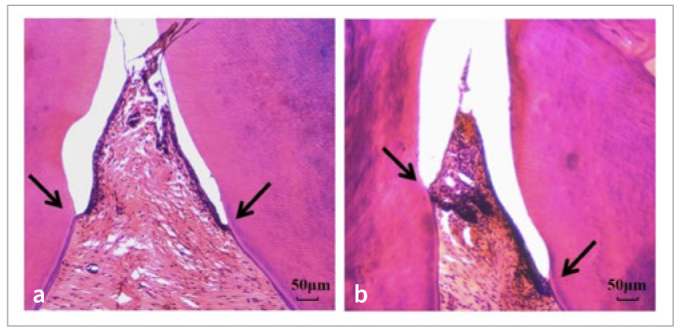
HE staining of periodontal tissue of maxillary second molar (100X original magnification). A: blank group; B: periodontitis group. The arrows point to the conjunctive epithelium of the maxillary second molar.

In the second batch of rats in group A, there was no redness or swelling of the gingiva, no tooth mobility, and no evidence of periodontal pockets in the maxillary second molars. However, compared to the initial modeling stage, groups B, C, and D showed red and swollen gingiva, bleeding upon probing, tooth mobility in a buccolingual direction, and the presence of periodontal pockets in the maxillary second molars (Fig 8). 3D reconstruction analysis using Hiscan Analyzer software revealed that, compared to group A, the modeling groups (B, C, and D) exhibited a certain degree of alveolar bone resorption in the area of the maxillary second molars (Fig 9). Decalcification, embedding, sectioning, and staining observations showed that, compared to group A, the modeling groups (B, C, and D) exhibited a statistically significant infiltration of inflammatory cells and epithelial proliferation towards the root on the side where the maxillary second molars were ligated (Fig 10).

### Determination of SIgA Type Antibody and CAMP Level

**Fig 11:**
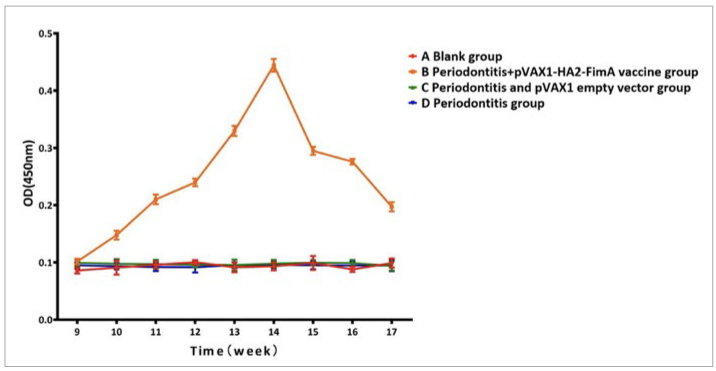
Trend of the level of anti-HA2 SIgA antibody in saliva.

**Fig 12:**
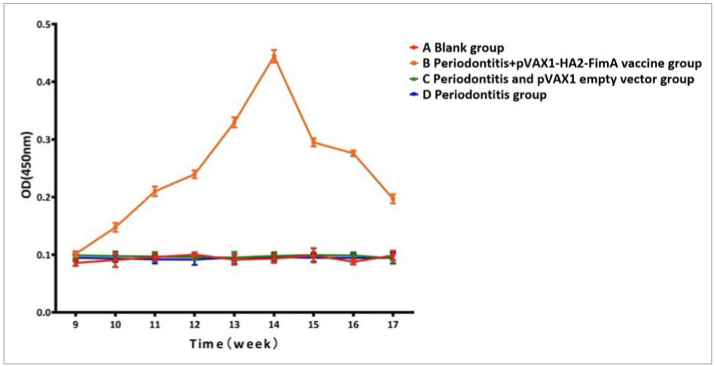
Trend of the level of anti-FimA SIgA antibody in saliva.

Before immunisation, there was no statistically significant difference in the levels of anti-HA2 SIgA antibodies and anti-FimA SIgA antibodies in the saliva of the four rat groups (p>0.05). After immunisation, there was a rapid increase in the levels of anti-HA2 SIgA antibodies and anti-FimA SIgA antibodies, reaching peak levels at week 14 and then slowly declining. Among them, group B (periodontitis + pVAX1-HA2-FimA group) had consistently higher antibody levels than the other three groups during weeks 10-17 (Figs 11 and 12), and the difference was statistically significant (p < 0.05).

**Fig 13:**
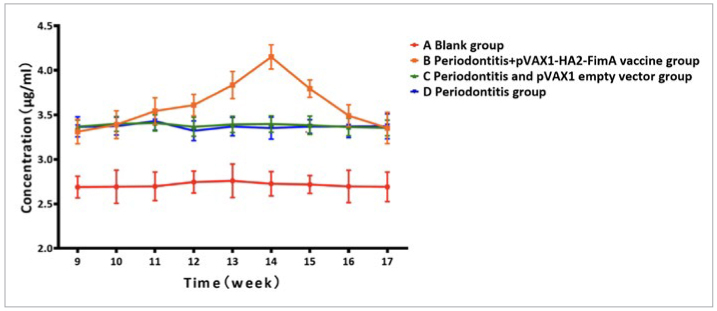
Trend of antimicrobial peptides in the saliva of rats.

The rat antimicrobial peptide ELISA kit results showed that the CAMP level trend in group B (periodontitis + pVAX1-HA2-FimA group) was similar to the levels of specific SIgA antibodies. After intranasal immunisation, there were changes in CAMP levels in rat saliva. CAMP levels gradually increased from week 9 to week 14 and then gradually decreased after reaching a peak at week 14 (Fig 13). Among them, during weeks 12 to 15, group B (periodontitis + pVAX1-HA2-FimA group) consistently had higher levels of CAMP in saliva compared to the other groups, and the difference was statistically significant (p < 0.05).

### Observation of Alveolar Bone Resorption in the Maxillary Second Molar of Rats

**Fig 14:**
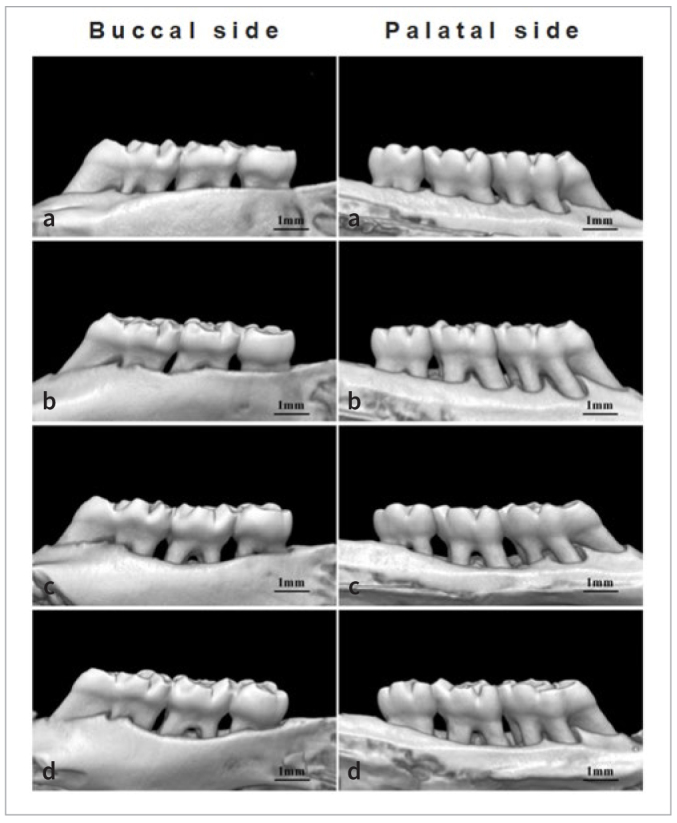
Micro-CT 3D reconstruction of the maxilla. a: blank group; b: periodontitis model + pVAX1-HA2-FimA group; c: periodontitis model + empty vector group; d: periodontitis model group.

**Fig 15:**
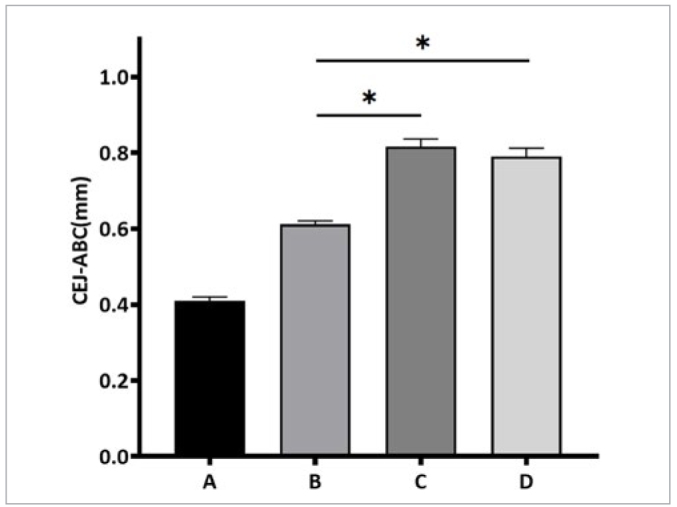
Alveolar bone resorption statistics. A: blank group; B: periodontitis model + pVAX1-HA2-FimA group; C: periodontitis model + empty vector group; D: periodontitis model group. *Statistically significant, p < 0.05.

Using Hiscan Analyzer Software, linear measurements were taken of the distance between the enamel-dentin junction and the alveolar crest at six sites (buccal and palatal sides) of the maxillary second molar (mesial, central, distal). A larger distance indicates greater alveolar bone resorption. The results showed that group A had no statistically significant alveolar bone resorption. Group B had less alveolar bone resorption compared to groups C and D, but group B had more alveolar bone resorption compared to group A. This difference was statistically significant (p < 0.05). There was no statistically significant difference in alveolar bone resorption between groups C and D (p > 0.05) (Figs 14 and 15).

## Discussion

Periodontal disease, as a chronic inflammatory condition, can cause damage to the periodontal tissues and resorption of the alveolar bone.^14^ Due to the initial asymptomatic nature of periodontitis, it often goes unnoticed by patients. Prolonged chronic inflammation can lead to irreversible destruction of the periodontal tissues, ultimately resulting in tooth mobility and tooth loss.^13^ Currently, non-surgical or surgical treatment methods are primarily used in clinical practice to manage periodontal disease, but recent research has shown that approximately 14% of patients have unsatisfactory outcomes after treatment.^7^ Subgingival microorganisms play a significant role in the effectiveness of periodontal therapy, as they cannot be eradicated solely through mechanical debridement and periodontal surgery. Appropriate adjunctive antimicrobial therapy can help reduce or inhibit the pathogens that have invaded the periodontal tissues.^25^

Due to the limitations of current periodontal treatments and the increasing antibiotic resistance of periodontal pathogens, there is a need to develop more effective adjunctive treatment methods. Host inflammatory response is a major driving factor in tissue destruction, so adjunctive therapies for periodontal disease should focus on modifying the host immune response.^23^ Periodontal vaccines have advantages in long-term prevention and treatment compared to short-term host immune modulation therapies such as lipoxin mediators, complement inhibitors, and cytokines.^20^ *P. gingivalis*, a gram-negative bacterium primarily residing in the periodontal pocket, can evade immune system detection through various virulence factors, leading to dysbiosis in the oral microbiota.^4^ Some studies have used *P. gingivalis* fimbriae, lipopolysaccharides, gingipains, and capsules as vaccine antigens, achieving varying degrees of success.^22^ Compared to other adjunctive treatment approaches, periodontal vaccines can promote pathogen clearance, block certain virulence factors, and shift the host immune response from destructive inflammation to a controlled immune homeostasis.^26^ However, DNA vaccines have the potential to trigger an autoimmune response or face a plethora of problems associated with genome integration.

An effective periodontal vaccine needs to elicit an immune response in the host against the pathogenic bacteria, with SIgA antibodies playing a crucial role in mucosal epithelial protection and immune regulation.^12^ Consistent with expectations, intranasal immunisation of rats resulted in detectable levels of specific SIgA antibodies in their saliva. The levels of CAMP in saliva also showed a similar trend to specific SIgA antibodies. However, the concentration of CAMP in the saliva of the control group rats remained lower than that of the other groups. This trend is consistent with clinical research findings that chronic periodontitis patients have higher concentrations of antimicrobial peptide LL-37 (cathelicidin LL-37) in their saliva and gingival crevicular fluid compared to healthy subjects.^11^ CAMP is the murine ortholog of LL-37 and exhibits similar antimicrobial activity against pathogens in vitro.^3^ Although the concentration of CAMP in rat saliva after intranasal immunisation was lower than the minimum bactericidal concentration for periodontal disease pathogens, CAMP plays a critical role in oral homeostasis. Studies have shown that CAMP provides a suitable microenvironment for angiogenesis, promotes the differentiation and migration of mesenchymal stem cells, inhibits osteoclast formation, and induces bone regeneration.^17,18^

In this study, we explored the mechanism of the periodontal gene vaccine based on transcriptomic sequencing. Our results showed a similar trend between CAMP levels and SIgA antibody changes, with increased saliva levels after immunisation. However, CAMP concentration was insufficient to eliminate periodontal disease pathogens. Further research using 16S rRNA gene sequencing technology is needed to investigate the subgingival microbial community from the perspective of oral ecological balance and further elucidate the mechanism of action of the periodontal gene vaccine. Additionally, this study did not include inflammation markers associated with periodontal disease. Subsequent research can further examine changes in the levels of inflammatory factors such as interleukin-6, interleukin-8, interleukin-17, and tumor necrosis factor-alpha in serum. Based on the results of this study, future experiments will be able to develop more comprehensive and effective periodontal vaccines.

## Conclusion

The HA2-FimA DNA vaccine can increase the levels of SIgA and CAMP in an experimental rat model of periodontitis, effectively activating the immune response and reducing alveolar bone loss.
